# Recurring cycles of deprivation of serum and migration in confined spaces augments ganglioside SSEA-4 expression, boosting clonogenicity and cisplatin resistance in TNBC cell line

**DOI:** 10.1038/s41598-025-99828-6

**Published:** 2025-05-14

**Authors:** Zain Nofal, Philipp Malakhov, Margarita Pustovalova, Nawar Sakr, Sergey Leonov

**Affiliations:** 1 Institute of Future Biophysics, Moscow Institute of Physics and Technology, MIPT, Phystech, Dolgoprudny, Russia 141701; 2 Federal Research Center for Innovator and Emerging Biomedical and Pharmaceutical Technologies, Moscow, Russia 125315; 3 Institute of Cell Biophysics of Russian Academy of Sciences, Pushchino, Russia 142290

**Keywords:** Confined migration, Breast cancer, Stem cell markers, Cisplatin resistance, Nano-particle endocytosis, Breast cancer, Cancer stem cells, Metastasis

## Abstract

The remarkable biophysical properties of metastatic migrating cells, such as their exceptional motility and deformability, enable them to migrate through physical confinements created by neighboring cells or extracellular matrix. This study explores the adaptive responses of breast cancer (BC) cell sublines derived from the highly aggressive, metastatic triple-negative MDA-MB-231 and the non-metastatic MCF7 human BC cell lines, after undergoing three rounds of confined migration (CM) stress. Our findings demonstrate that CM elicits common and cell-type specific adaptive responses in BC cell sublines. In particular, both cell sublines exhibit a similar enhancement of clonogenicity and nanoparticle (NP) uptake activity, indicating tumorigenic potential. We have, for the first time, shown that stimulation with CM induces a hybrid epithelial-to-mesenchymal transition (EMT) phenotype of MDA-MB-231 cells. This transition is characterized by a significant rise in the expression of stage-specific embryonic antigen-4 (SSEA4), alongside a substantial decline in the population of CD133+ cells and a marked reduction in Ki67 expression in the MDA-MB-231-derived subline following Cis-Platin treatment. These changes are likely associated with heightened resistance of this subline to cisplatin. In contrast, CM induces far fewer such alterations in the MCF7-derived counterpart with a notable increase of CD133+ population, which seems to be insufficient to change cell susceptibility to cisplatin exposure. This study contributes to our understanding of the adaptive mechanisms underlying metastasis and drug resistance in breast cancer, emphasizing the need for personalized approaches in cancer treatment that consider the heterogeneous responses of different cancer subtypes to environmental stresses.

## Introduction

Metastasis remains the primary cause of cancer-related deaths, accounting for approximately 90% of cancer mortality^1^. Metastasis is a multifaceted process that unfolds through several intricate stages: local invasion, intravasation, survival in the bloodstream, extravasation, and colonization of distant sites^2^. Throughout this journey, cancer cells face and must overcome diverse micro-environmental challenges, such as navigating narrow passages created by adjacent cells, the extracellular matrix, and blood vessels^2^. Recent studies have highlighted the importance of mechanical cues in cancer cell behavior and metastasis^3^. Confined migration (CM), in particular, has been shown to induce significant changes in cancer cell phenotype and function^4^. However, the long-term effects of repeated CM on cancer cell populations and their adaptive responses remain poorly understood.

Breast cancer (BC), characterized by its diverse molecular subtypes, serves as an excellent model for examining how various cancer cells adapt differently to environmental stresses^5^. The MDA-MB-231 cell line, known for its aggressive triple-negative status and high metastatic potential, alongside the non-metastatic, estrogen receptor-positive MCF7 cell line, exemplifies two significant subtypes of breast cancer, each showcasing unique clinical attributes^6^.

Cancer stem-like cells (CSCs) have been implicated in metastasis, drug resistance, and tumor recurrence^7^. CD133, also known as prominin-1, and stage-specific embryonic antigen-4 (SSEA4), both were recognized as potential CSC markers, and have been linked to stemness and chemoresistance in a variety of cancers, including BC^8,9^. However, the variability in the expression of these markers and their functional relevance in how different cancer subtypes respond to environmental stressors is still not well understood.

The epithelial-to-mesenchymal transition (EMT) is a crucial process in cancer progression and metastasis^8^. Alterations in EMT markers can reveal shifts in cellular plasticity and invasive capacity. Moreover, drug resistance continues to pose a significant challenge in cancer chemotherapy, with the development of chemoresistance through EMT activation emerging as a crucial focus of research^9^. Diversity among genotypically similar cancer cells, whether in vitro or in vivo, primarily arises from two key factors: cell cycle phase and overall proliferative rate^10,11^. For decades, these factors have been strongly associated with sensitivity to standard chemotherapy treatments^12,13^. By investigating how CM influences EMT status, cell cycle phase and overall proliferative rate across various breast cancer subtypes, we can uncover valuable insights into the mechanisms driving pro-metastatic adaptation and chemoresistance.

Recent research has highlighted the significant role of disrupted endocytic activity in the malignancy of cancer cells^10^; however, there is limited understanding of how CM-induced stress influences endocytosis in BC cells.

In this study, we investigate the adaptive responses of MDA-MB-231 and MCF7 cells undergoing several rounds of CM. We examine changes in stem-like cell and EMT markers, along with cisplatin resistance, accompanied by alterations in proliferation, cell cycle progression and endocytic activity, to comprehend the cellular processes that are acquired after CM process. Our findings may have significant implications for understanding the mechanisms of cancer cell adaptation during metastasis and for developing new strategies to target metastatic cells. Through the analysis of responses from two breast cancer cell lines with varying metastatic propensities, we aspire to illuminate the diversity of adaptive mechanisms in solid tumors and underscore the importance of tailored, subtype-specific strategies for cancer treatment.

## Results

### CM sublines have higher self-renewal ability than their parental cell lines

**Fig. 1:**
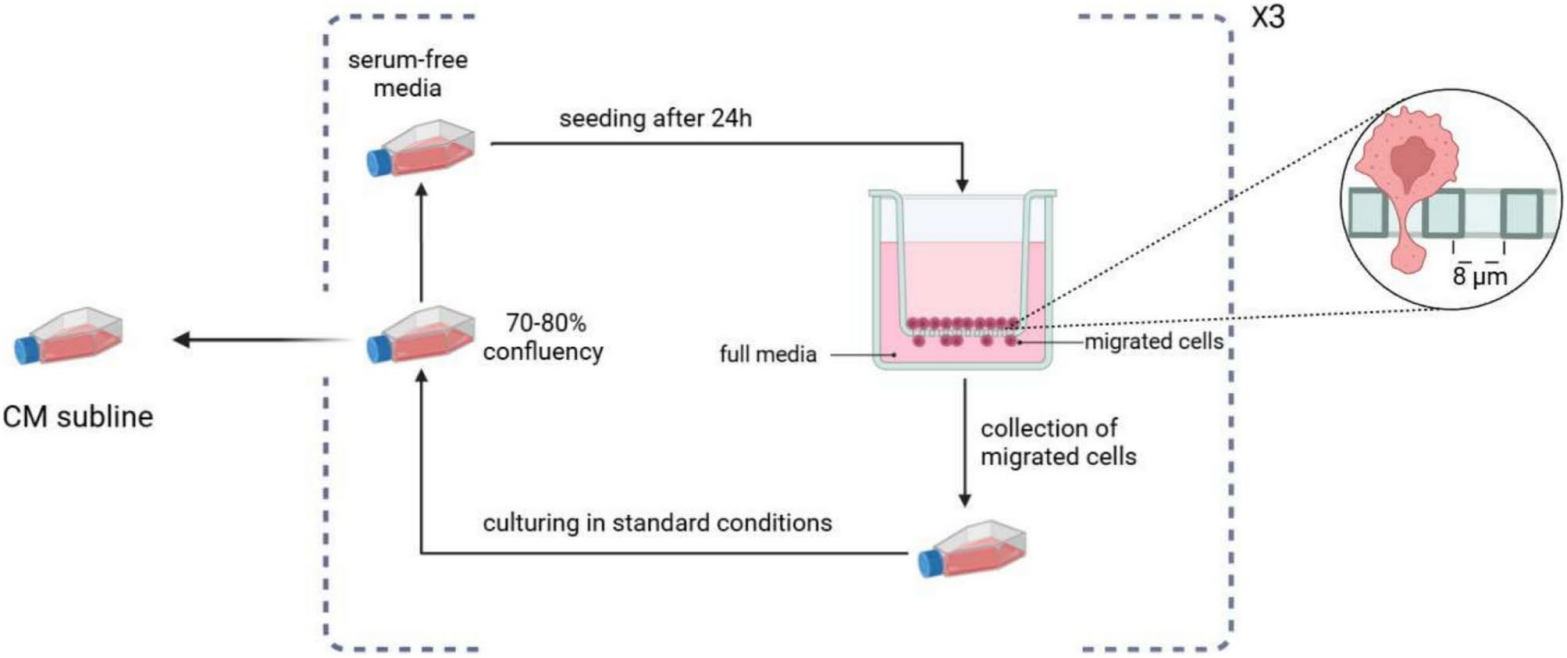
a schematic representation of the process of obtaining the Confined Migration (CM) sublines. Cells were starved for 24h before seeding them into an 8 µtranswell insert containg full media in the bottom chamber. migrated cells were collected from the bottom chamber and the process was repeated for a total of 3 times.

**Fig. 2:**
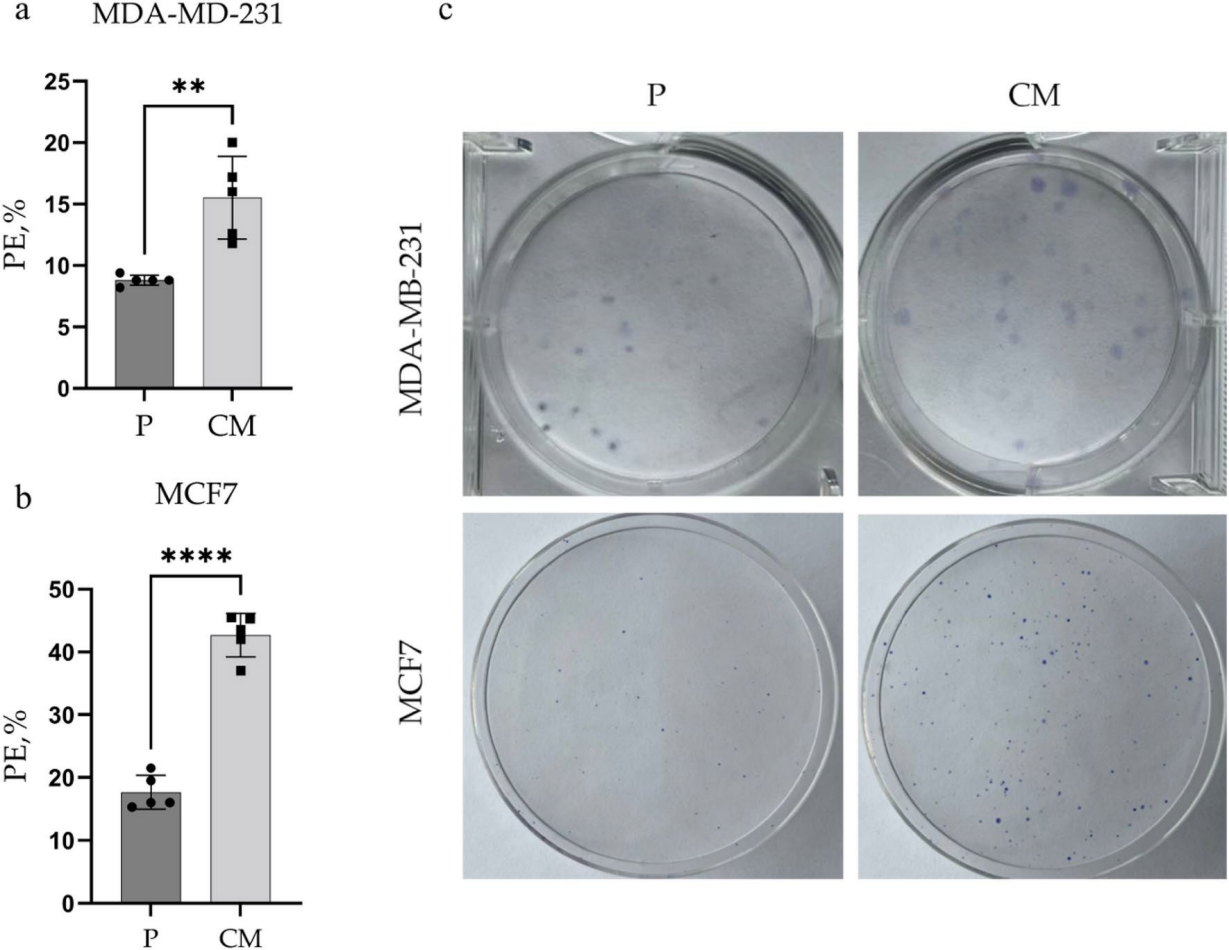
Clonogenic assay under anchorage-dependent conditions. (**a**) and (**b**), the plating efficiency (PE, %) of both MDA-MB-231 and MCF7 cell lines before (P, parental) and after (CM) confined migration; (**c**) Representative images of Giemsa-stained colonies of MDA-MB-231 and MCF7 cells. Data are means ± SEM of three independent experiments. **$p<$0.01; ****$p<$0.0001.

The Confined Migration (CM) sublnies were obtained after 3 rounds of migration through an 8 µm transwell membrane (Fig. 1). The resulting sublines were designated as the CM sublines for each respective parental (P) cell line. Clonogenic anchorage-dependent growth assay is widely used to evaluate the ability of individual cells to proliferate and give rise to colonies, where a higher plating efficiency indicates enhanced survival and tumorigenicity in vivo^14^. As shown on Fig. 2, the CM sublines of MDA-MB-231 and MCF7 cells, had a 1.76-fold (*p*-value <0.01) and 2.41-fold (*P*-value <0.0001) higher plating efficiency compared to their parental counterparts.

### Confined migration consistently upregulates the nanoparticle (NP) endocytosis

To further investigate the phenotypic changes induced by serial confined migration, we examined the uptake of fluorescent carboxylated NPs in both parental and CM sublines of MDA-MB-231 and MCF7 cells. This experiment is particularly relevant as increased endocytic activity has been associated with enhanced malignancy and metastatic potential in cancer cells reference^15^ The uptake of NPs can serve as a functional readout of the cells’ endocytic capacity, which is often altered in more aggressive cancer phenotypes.

**Fig. 3:**
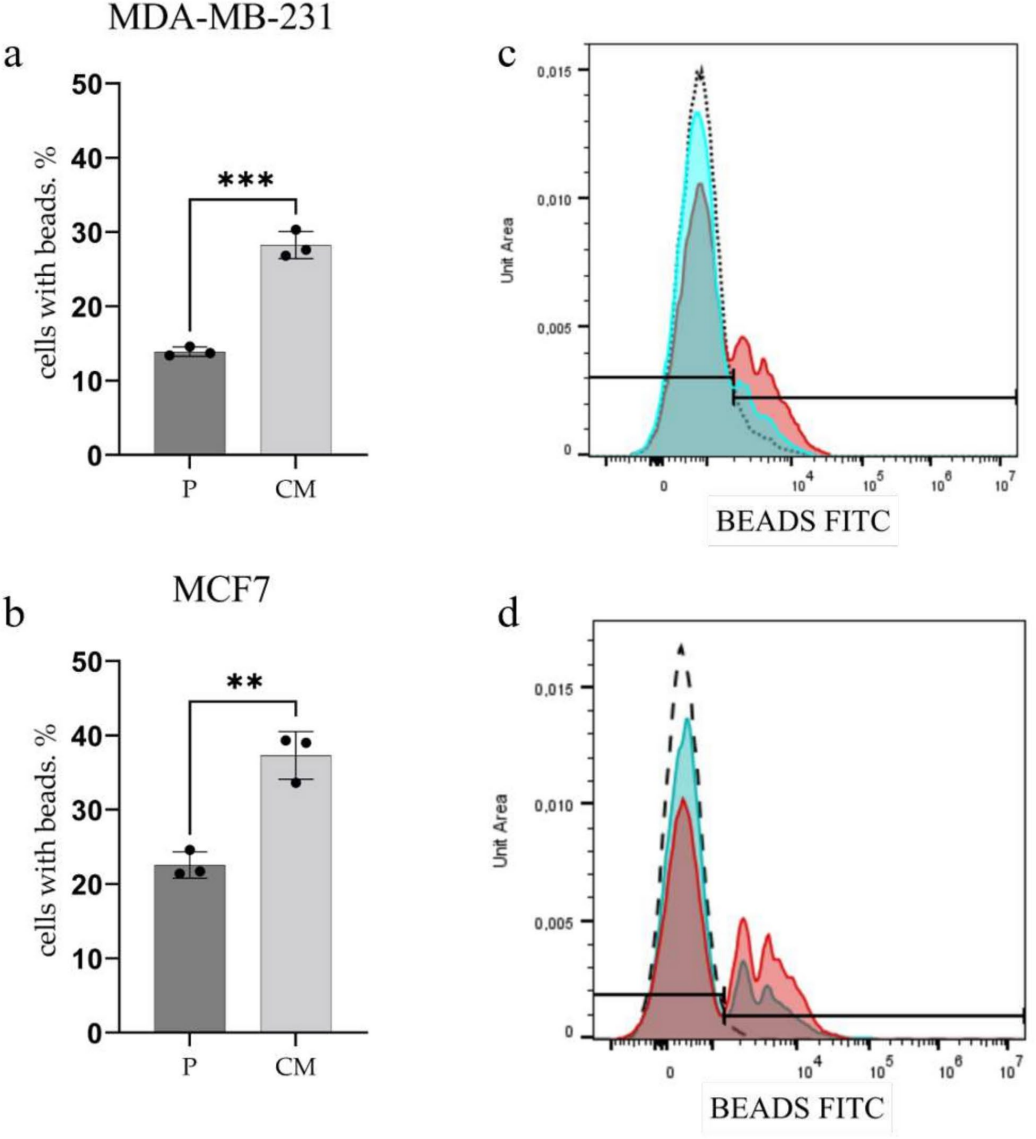
Flow cytometry analysis of nanobeads endocytosis. a and b, fractions of both MDA-MB-231 and MCF7 cells engulfing 200nm carboxylated nanobeads before (P, parental) and after (CM) confined migration. c and d, flow cytometry histograms representing the nanobead uptake signal (FITC channel) in MDA-MB-231 and MCF7 cells, respectively. Bead uptake by parental (P) cells are shown in a blue color histogram and CM cells are shown in a red color histogram. Data are means ± SEM of three independent experiments. ***p<0.001.

Our results reveal that confined migration consistently increase in NP uptake by both cell sublines, suggesting a common adaptive response that may contribute to their enhanced clonogenic properties (Fig. 3 ). MCF7CM cells demonstrated 1,65-fold increase (*p* < 0.01), while MDA-MB-231CM cells showed more prominent 2-fold increase (*p* < 0.001) in uptaking NPs compared to parental cells. This consistent elevation in NP uptake across both cell lines is particularly noteworthy, as it represents one of the few changes that are uniformly observed in both MCF7 and MDA-MB-231 cells after serial migration rounds, alongside the observed increase in plating efficiency (Fig. 2). The enhanced endocytic activity, as indicated by increased NP uptake, may contribute to the cells’ ability to interact with and adapt to their microenvironment more effectively.

### A hybrid EMT phenotype identified in MDA-MB-231CM, while absent in MCF7CM subline

**Fig. 4:**
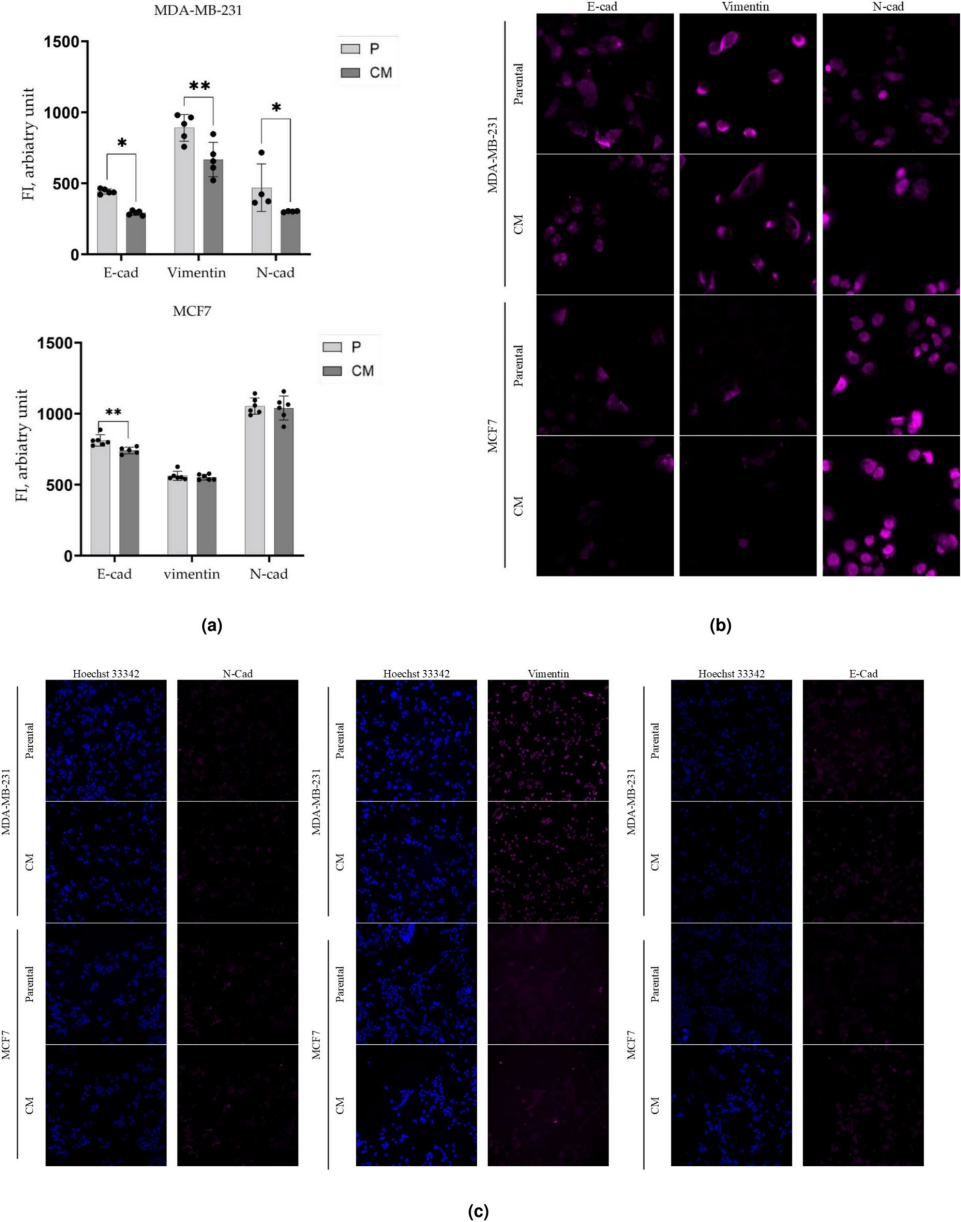
Expression of EMT Markers. The integrated fluorescence intensities (FI) of N-Cadherin, E-Cadherin, and Vimentin expression before (parental, P) and after confined migration (CM) in MDA-MB-231 and MCF7 cells (**a**). (**b**), zoom-in insets of representative fluorescence images showing staining of the EMT markers. (**c**) Representative fluorescence images showing staining of the EMT markers in magenta and the nuclear stain in blue at 10x objective **p<0.01 *p<0.05.

To investigate whether the enhanced clonogenicity of CM cells is associated with changes in their EMT phenotype, we investigated the expression of N-Cad, E-Cad, and Vimentin, three key EMT markers, using single-cell high content immunofluorescence imaging and analysis. MDA-MB-231CM cells showed a significant decrease in all three markers: E-, N-Cadherins and Vimentin - by almost 35% (*p*-value<0.05), 20% (*p*-value<0.01) and 25% (*p*-value<0.05), respectively, compared to the parental cells (Fig. 4a). MCF7CM cells showed no significant change in neither N-Cad nor Vimentin expression. However, E-Cadherin demonstrated a slight but significant (*p*-value<0.01) decrease by 8.73% compared to the parental cell line. Representative zoom-in insets and fluorescence images for all three markers are shown in Fig. 4b,c. These findings align with recent studies highlighting the role of hybrid EMT phenotypes in enhancing metastatic potential in cancer cells^16^, proliferative capacity^17^, and aggressiveness of breast cancer cells^18^.

### CM modulates the CSC marker profile of BC cell lines

Stage-specific embryonic antigen-4 (SSEA-4) is a glycosphingolipid that is predominantly overexpressed in certain cancers^19,20^, and its presence has been associated with the progression of breast cancer, in particular SSEA-4 has emerged as an innovative marker for identifying diverse and invasive subpopulations of tumor cells^21^ . Furthermore, elevated expression of cell-surface SSEA-4 is linked to diminished cell-cell interactions and an enhanced migratory phenotype in prostate cancer cells. This indicates that SSEA-4 plays a crucial role in cancer invasion by affecting the adhesion of cells to the extracellular matrix.

**Fig. 5:**
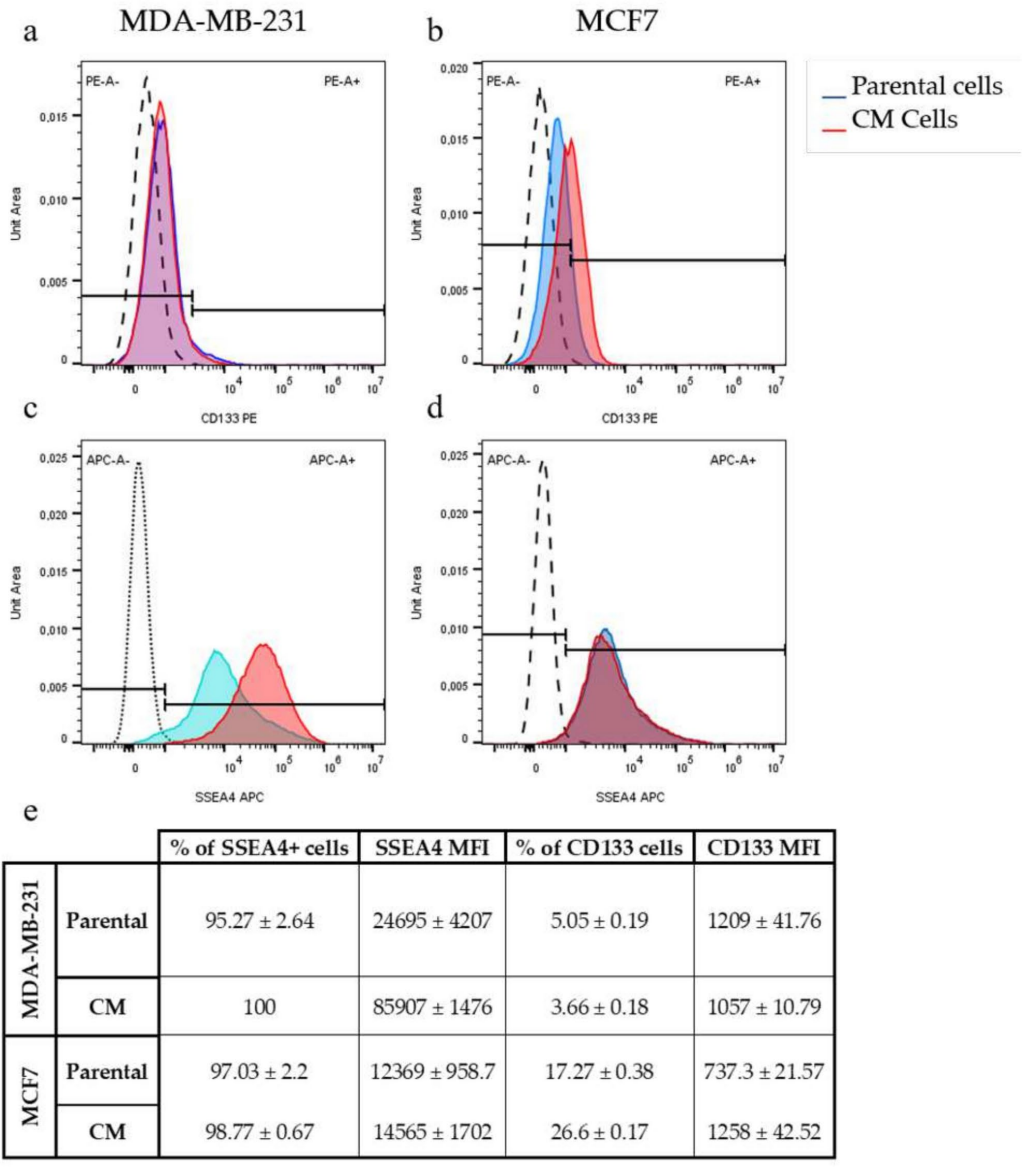
The flow cytometry analysis of the expression of surface stem-like cell markers in BC cells. Flow cytometry histograms representing the expression of CD133 (**a**, **b**) and SSEA4 (**c**, **d**) on MDA-MB-231 and MCF7 before (parental,P), and after (CM) confined migration. (**e**) a table depicting the mean fluorescent intensities (MFI) of CD133- and SSEA4-positive fractions of MDA-MB-231 and MCF7 cells.

Both parental cell lines exhibited similar fractions and levels of expression of SSEA4 as shown in Fig. 5e However, the MDA-MB-231CM cells exhibited over a threefold (MFI fold increase=3.479 ± 0.6, *P*-value < 0.0001) increase in SSEA-4 expression relative to the parental population.

In contrast, MCF7CM cells exhibited a negligible increase in SSEA-4 expression (MFI fold increase = 1.178 ± 0.14, *p*-value=0.1234), suggesting that their cell-cell interactions remain unchanged.

CD133, also known as prominin-1, is a pivotal five-transmembrane domain cell-surface glycoprotein that serves as a key biomarker for the identification and isolation of a particular subpopulation referred to as “cancer stem-like cells” across various neoplasms, including breast cancer^22^. CD133+ cells exhibit stemness characteristics such as resistance to drugs and radiation, the ability to self-renew, and differentiation potential^23^. Additionally, they demonstrate high proliferation rates and possess the capability to form tumors in xenograft models^24^.

Our results reveal divergent fractions of CD133-positive cells between both parental and CM sublines of MDA-MB-231 and MCF7 cells using flow cytometry. The parental MDA-MB-231 cells demonstrated a markedly reduced fraction of CD133+ cells, 5.05 ± 0.19% in contrast to their MCF7 counterparts, which exhibited 17.27 ± 0.38% prevalence (Fig. 5e). The proportion of CD133+ cells experienced a notable decrease in MDA-MB-231CM cells, with a mean of 3.66% ± 0.18% (*p*-value < 0.001) when compared to the parental cells. In contrast, the MCF7CM subline exhibited a significant increase in the fraction of CD133+ cells, reaching a mean of 26.60 ± 0.17% (*p*-value < 0.0001) relative to the parental population.

### Confined migration augments resistance to cisplatin (CPT) in MDA-MB-231CM, but not MCF7CM cells

**Fig. 6:**
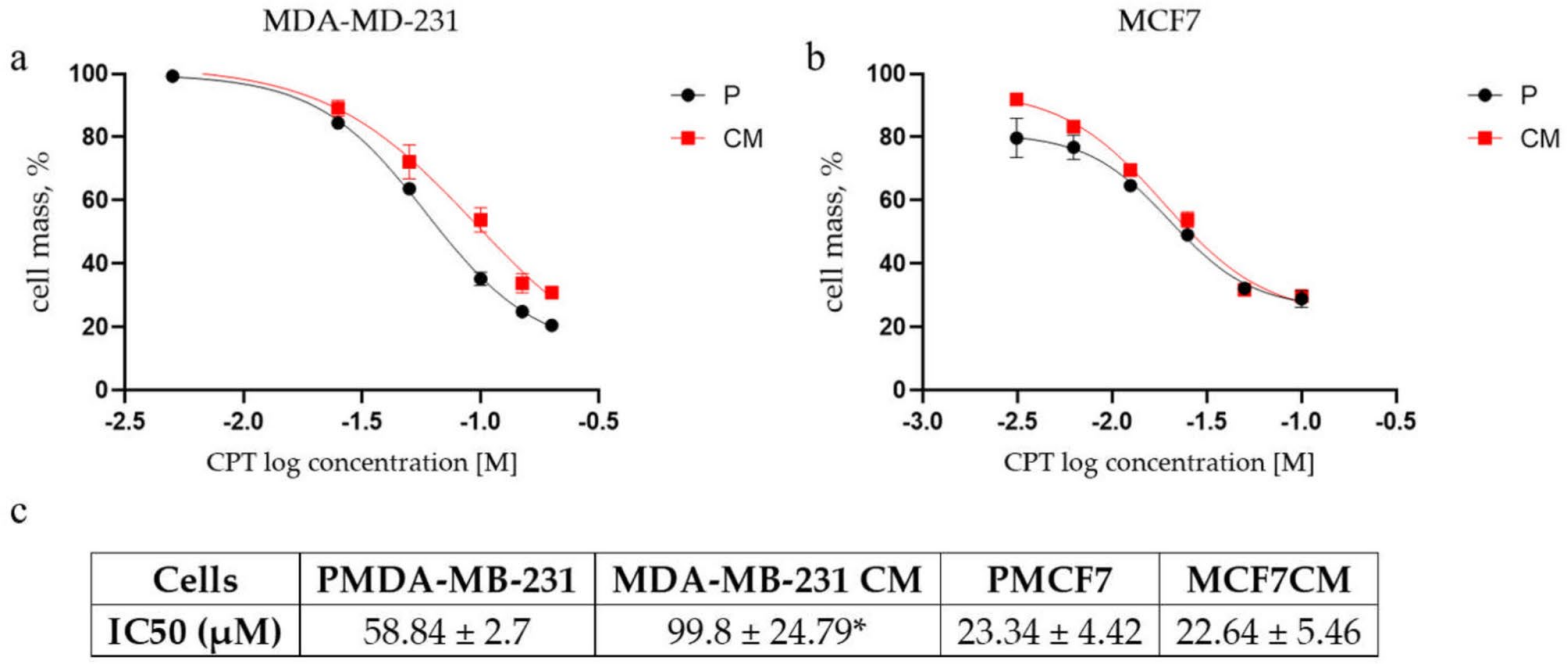
Dose response effect of cisplatin in MDA-MB-231 and MCF7 cells. a and b, Cisplatin dose-dependent survival curve of MDA-MB-231 (**A**) and MCF7 (**B**) cells before (parental, P) and after (CM) confined migration measured by SRB assay. (**c**) Table representing IC50 values calculated by GraphPad software. *p<0.05.

Cisplatin demonstrates significant efficacy as a standalone treatment in the initial management of metastatic breast cancer^25,26^. Given the observed divergent expression of SSEA-4 and CD133-two critical markers associated with cancer progression and chemoresistance-we conducted an analysis of the dose-response survival rates of the CM sublines compared to their parental lines, 48 hours after treatment with CPT. Parental MCF7 cells exhibited significantly greater sensitivity to CPT than MD-MB-231 cells, as evidenced by their respective IC50 values: 23.34± 4.42 µM for MCF7 and 58.84 ± 2.7 µM for MD-MB-231 (Fig. 6c). The MDA-MB-231CM cells displayed a significantly higher IC50 (99.80 ± 24.79 µM) in comparison to their parental counterparts, highlighting an increased resistance to CPT (Fig. 6a). In comparison, the MCF7CM cells exhibited no notable difference in IC50 for CPT when analyzed alongside their parental counterparts, registering value of 22.64 ± 5.46 µM (Fig. 6b).

### Confined migration alters CPT-induced heterogeneous cell fate choices in BC cell lines

Cisplatin, a potent DNA-damaging agent, effectively inhibits cell proliferation by triggering both cell cycle arrest and cell death^27,28^. Considering the notable resistance to CPT treatment in the MDA-MB-231CM subline, it became essential to explore the roles of cell cycle arrest along with programmed cell death in shaping the proliferation curves triggered by this chemotherapeutic agent.

**Fig. 7:**
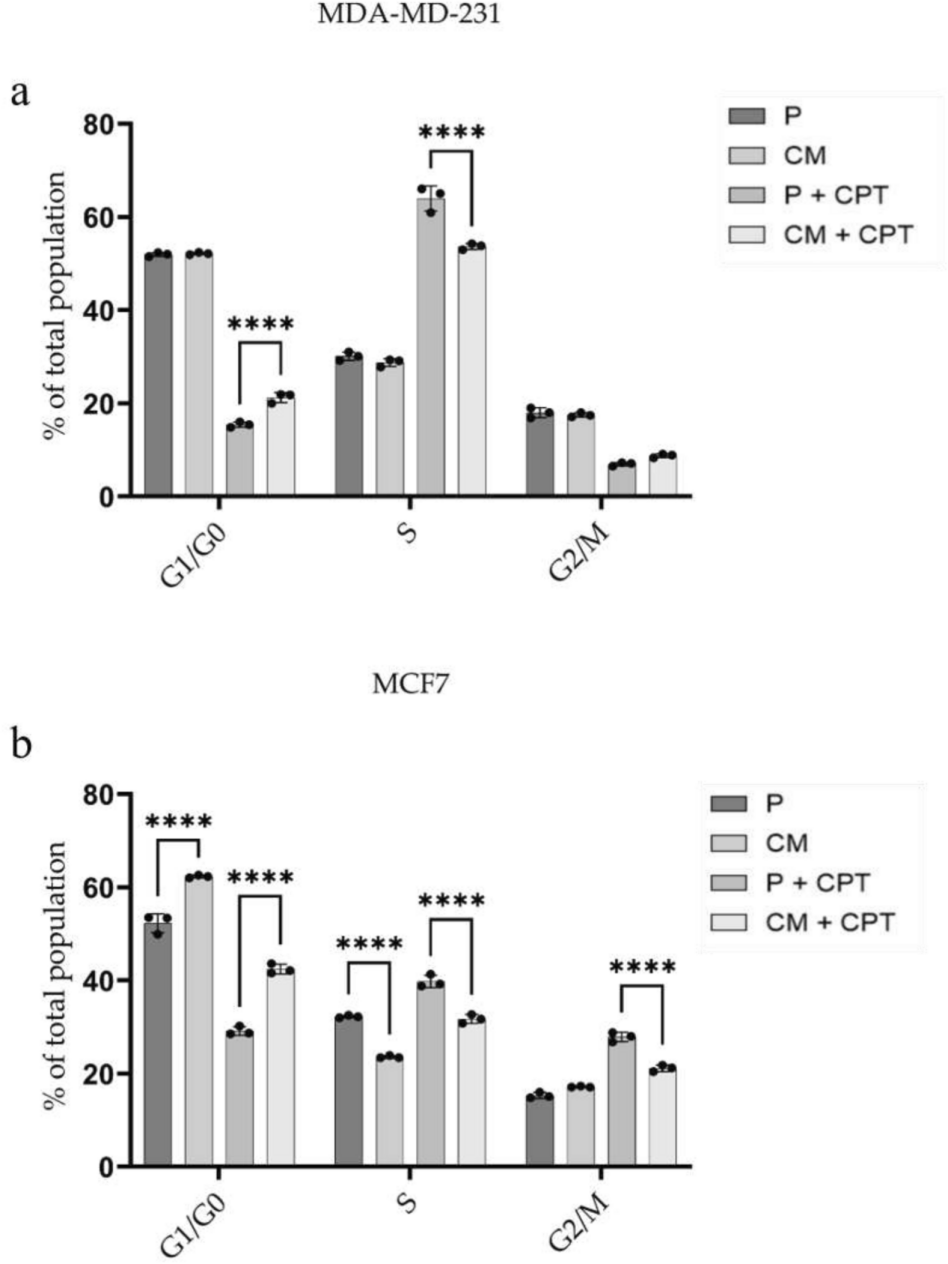
Cell cycle distribution of MDA-MB-231 and MCF7 cells with (+CPT) and w/o cisplatin treatment. Flow cytometric analysis of cell cycling of parental (P) and confined migration-selected (CM) MDA-MB-231 (**a**) and MCF7 (**b**) cells at 48 hours post-treatment with IC50 of cisplatin (+CPT). ****p<0.0001.

First, we assessed cell cycle progression by employing propidium iodide (PI) staining analyzed through flow cytometry. Both parental cell lines exhibited almost equal distribution of cells in each phase of cell cycle. Confined migration did not lead to any notable changes in the cell cycle progression of MDA-MB-231CM cells: with 51.9% in G1-G0 , 29.1% in S, and 17.4% in G2-M phases (Fig. 7a). In contrast, it significantly increases the fraction of MCF7CM cells in G1-G0 phases (from 52.3 up to 62.3%) on expense of cells in S-phase (from 29.1 down to 23.4%, Fig. 7b).

**Fig. 8:**
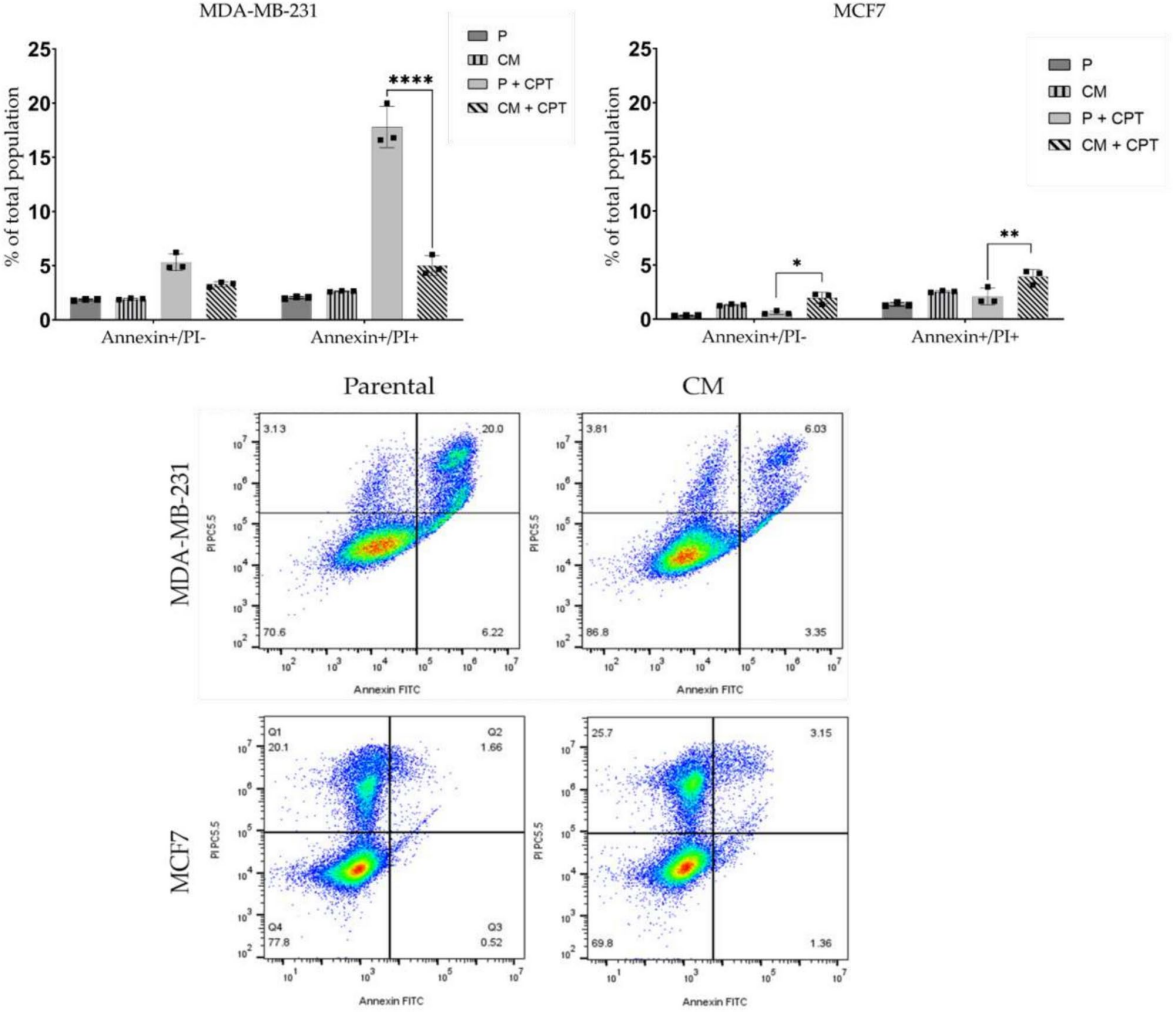
Analysis of the nature of cell death in MDA-MB-231 and MCF7 Cell Lines Following cisplatin Treatment. (upper panel) Flow cytometric analysis of apoptotic (Annexin V+/PI−) and necroptotic (Annexin V+/PI+) fractions of parental (P) and confined migration-selected (CM) MDA-MB-231 and MCF7 cells at 24 hours post-exposure to IC50 of cisplatin (+CPT). (lower panel) Representative Scatter plots of the Annexin/PI staining after CPT treatment. *p<0.05; ****p<0.0001.

48h after exposing breast cancer cell lines to CPT at concentrations around IC50, parental MDA-MB-231 cells demonstrate significant accumulation in S-phase from 29.2 up to 60.9%, while fraction of G1-G0 and G2-M cells were reduced from 52.3 down to 15.4%, and from 18 down to 7.08%, respectively. Under the same conditions, parental MCF7 cells increase the fractions of S-phase (up to 40%), and G2-phase (up to 30%) cells, while the percentage of cells in G1-G0 drastically decreased to an average of 28%. Confined migration reduces the extent of CPT impact on MDA-MB-231CM cells, attenuating their arrest in S-phase of cell cycle. Seemingly, CM also influence the CPT impact on MCF7CM cells, conferring their less prominent arrest in both S- and G2-phases of cell cycle. These results indicate that cell cycle changes in response to CPT are independent of their initial cell cycle distribution, which is in agreement with the classification of cisplatin as a cross-linking agent, generally considered to be a cell cycle-nonspecific agent. We next investigated whether cell cycle phase arrest also affects the final outcomes of CPT treatment, and how CM may interfere with cell fate decisions. Hence, we assessed nature of cell death by employing Annexin V and Propidium Iodide (PI) staining analyzed through flow cytometry. Confined migration did not lead to any notable changes in the apoptotic (Annexin V+/PI−), necroptotic (Annexin V+/PI+), or necrotic (Annexin V−/PI+) fractions of non-treated either MDA-MB-231CM or MCF7CM sublines when compared to their parental counterparts (Fig. 8). 24h after exposing breast cancer cell lines to CPT at concentrations around IC50, parental MDA-MB-231 cells demonstrate increase of early apoptotic and late apoptotic cells up to  5–6% and 16–20% (*p*<0.001), respectively. Compared to their CPT-treated parental counterparts, MDA-MB-231CM cells showed only the trend (3–4%) and significance in decrease (3–6%, *p*<0.0001) in the fraction of apoptotic and late apoptotic (necroptotic) cells, respectively, at the same time. CPT-exposed parental MCF7 cells demonstrated insignificant changes in the fractions of both early apoptotic ($\tilde{0}$.6%) and late apoptotic ($\tilde{2}$.1%) cells compared to their untreated counterparts. Compared to their CPT-treated parental counterparts, MCF7CM cells exhibited a significant increase in fractions of early apoptotic ($\tilde{1}$.95%, *p*<0.05) and late apoptotic ($\tilde{3}$.93%, *p*<0.01) cells (Fig. 8). These findings suggest that the CM-induced reduction in the effects of CPT on cell cycle distribution significantly influences cell fate. It increases the proportion of apoptotic and necroptotic MCF7CM cells while markedly reducing the fraction of necroptotic MDA-MB-231CM cells. The divergent cell fate decision observed in MDA-MB-231CM cells could potentially enhance their chances of survival, leading to a shift in the survival curve and suggesting an increase in their resistance to CPT treatment.

### Following migrating within a confined environment, MDA-MB-231 cells seemingly revoke their cell cycling in response to CPT treatment

Besides the distinct cell cycle phases, cells are characterized by a more ambiguous proliferative state that is thought to influence their reaction to chemotherapeutics^29,30^. Proliferative states are commonly assessed through immunohistochemical analysis of proliferation markers, including Ki67, with proliferation recognized for many years as an important prognostic clinical indicator^31^. Recent data indicates that precise quantification of Ki67 antibody staining provides valuable insights that extend beyond merely determining whether a cell is in a proliferative state. This technique effectively distinguishes between rapidly cycling cells, which experience brief periods of quiescence, and those slowly cycling cells that remain in quiescence for significantly longer durations before eventually re-initiating the cell cycle^32^.

**Fig. 9:**
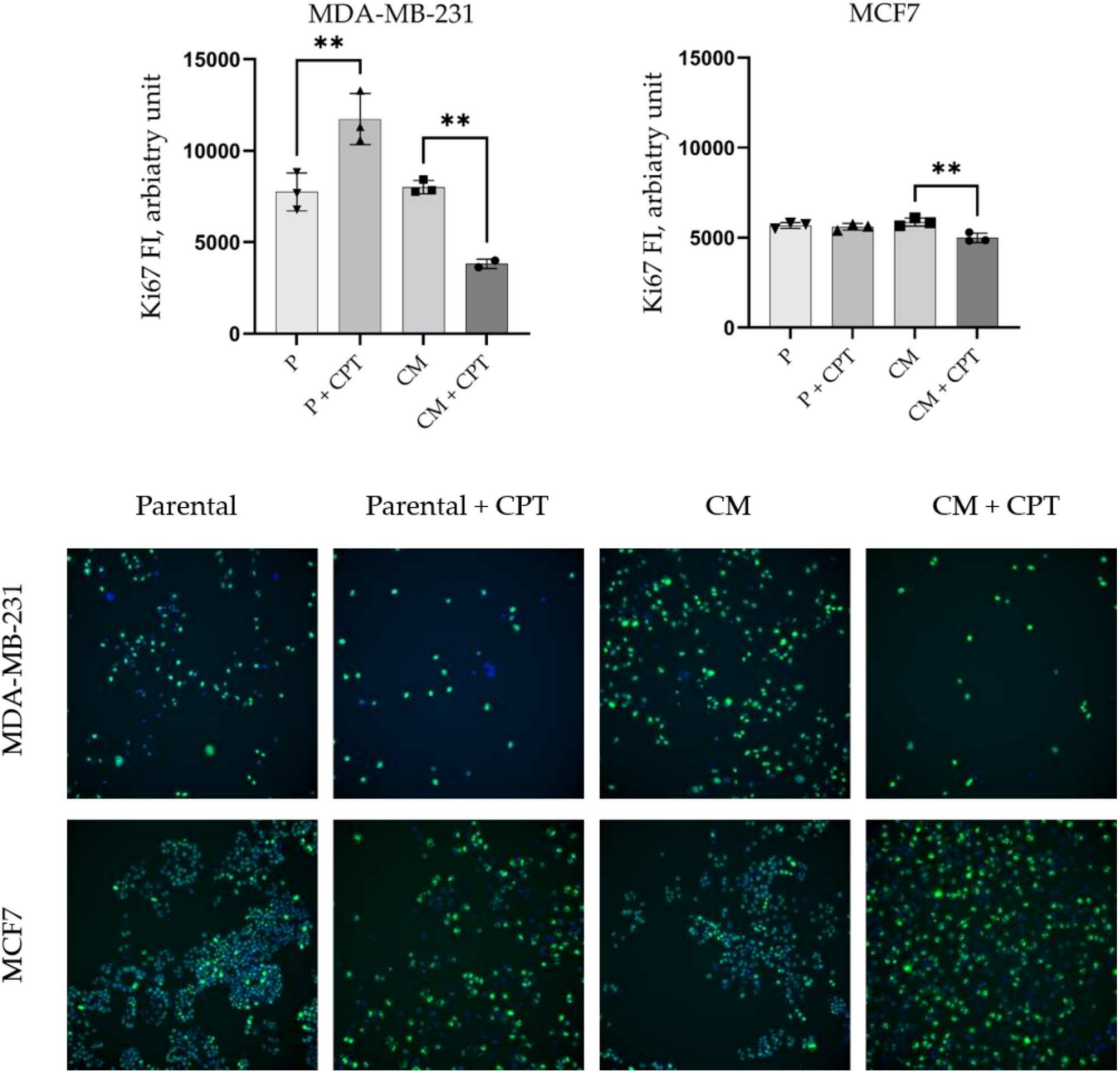
High-content imaging and analysis of Ki67 Expression in MDA-MB-231 and MCF7 Cell Lines Following Cisplatin Treatment. (upper panel), the integrated fluorescence intensities (FI) of Ki67 expression in parental (P) and confined migration-selected (CM) MDA-MB-231 and MCF7 cells at 24 hours post-treatment with IC50 of cisplatin (+CPT). (lower panel), representative images of nuclei (blue) and Ki67 (green) staining.

To assess the proliferative state of parental and CM cells, we performed immunofluorescent analysis of Ki67 expression. The relative fluorescence intensity (RFI) of Ki67 was quantified using single-cell high content imaging and analysis. Our results indicate that CM did not affect the proliferation state of neither MCF7CM nor MDA-MB-231CM cells compared to their parental counterparts (Fig. 9).

Subsequently, we examined whether the confined migration influence CPT effect on the proliferation state and the outcomes related to cell fate. Each cell line was treated with cisplatin for 24 hours, after which the drug was removed, and cells were allowed to recover in fresh medium for 48 hours before staining for Ki67. Non-treated cells were used as a control. CPT at concentrations around IC50 induces a significant increase 151.3% ± 18.1%, *p* < 0.001) in Ki67 expression in the parental MDA-MB-231 cells compared to their untreated counterparts (Fig. 9). Compared to their CPT-treated parental counterparts, MDA-MB-231CM cells exhibited a decrease in Ki67 expression following cisplatin treatment (49.17% ±3.35%, *p* < 0.001). This indicates that, while parental MDA-MB-231 cells exhibit increased cell cycling in response to cisplatin treatment, CM cells appear to enter a state of halted cycling, potentially transitioning into quiescence. Exposure of parental MCF7 cells to IC50 concentrations of CPT did not affect the the expression of Ki67. However, MCF7CM cells demonstrate a modest but significant decrease in Ki67 expression following cisplatin treatment (87.93% ± 4.64%, *p*<0.05).

## Discussion

The remarkable biophysical properties of metastatic migrating cells, encompassing their exceptional motility and deformation abilities, enable them to skillfully navigate the physical limitations posed by neighboring cells and the extracellular matrix (ECM)^33,34^. Solid tumors are affected by the mechanical forces of the surrounding tissue, which cause the tumor cells’ deformation. This has a significant impact on the behavior of the cells, including how they migrate, divide, and survive^35^. The main distinction between a confined migratory cell and a 2D cultured cell lies in the level of physical constraint. When cells migrate through physical constraints, their movement is confined by interactions with the surrounding structures. This microenvironment provides more contact points compared to cells in a 2D environment, where cells have the freedom to grow, multiply, and migrate with minimal physical restriction. Using the classical Boyden Chamber, we successfully isolated a fraction of cancer cells confined within a physically restricted microenvironment. The cells were permitted to migrate through a membrane featuring 8 um pores, which are smaller than their size range of 12-20 um, as they navigated a gradient of serum concentration established between the upper and lower chambers. In solid tumors, fluctuating nutrient availability, including serum deprivation, is a common feature of the microenvironment. Thus, while serum starvation may act as a confounding variable in controlled experimental systems, it also represents a physiologically relevant condition that influences tumor cell behavior. This dual role highlights the complexity of studying cancer cell migration and necessitates careful interpretation of our findings.

Here, we present evidence indicating that confined migration (CM) enhances the resistance of the TNBC cell line MDA-MB-231 to cisplatin. This phenomenon is linked to the development of a hybrid EMT phenotype, characterized by a significant increase in stage-specific embryonic antigen-4 (SSEA4) expression. Additionally, we observed a pronounced down-regulation of Ki67 expression and a reduced fraction of CD133+ cells. Comparing highly metastatic MDA-MB-231 and non-metastatic MCF7 cell lines, our study reveals that repetitive confined migration induces significant phenotypic and functional changes, with some alterations being cell-type specific while others are more common. Our findings provide insights into the adaptive mechanisms of cancer cells under migration stress and their potential implications for metastasis and drug resistance.

The increased plating efficiency observed in CM sublines of both MDA-MB-231 and MCF7 (Fig. 2) suggests an enhanced capacity for self-renewal. This is consistent with previous studies showing that migration through confined spaces can select for cells with increased tumorigenic potential^36^ and for cells having higher proliferation and DNA repair rates^37^. The observed consistent increase in nanoparticle uptake by both CM sublines (Fig. 3) also witnesses their augmented tumorigenic potential, following CM-induced stress. Recent studies have shown that increased endocytic activity is associated with cancer stem cell-like properties and may play a role in maintaining stemness^38^. Hence, the higher plating efficiency could be attributed to the activation of survival pathways or the selection of cells with stem-like properties during the migration process.

The enhanced endocytic capacity observed in both CM sublines may be linked to the acquisition of stem cell-like characteristics since it correlates with significant and marginal upregulation of SSEA4 expression (Fig. 5) in MDA-MB-231CM and MCF7CM cells, respectively. In turn, the upregulation of SSEA4 is accompanied by a significant (*p* < 0.05, *p* < 0.01) decrease in cell-to-cell contacts, as evidenced by a decrease in E-cadherin expression in MDA-MB-231CM and MCF7CM cells, respectively (Fig. 4). This finding supports similar SSEA4-E-cadherin relationships observed in prostate cancer cells^39^. Interestingly, we observed a hybrid EMT phenotype in MDA-MB-231CM cells, characterized by decreased expression of E-cadherin, N-cadherin, and vimentin. In our study, we intentionally did not analyze the co-expression of epithelial and mesenchymal markers in the same cell. Instead, we accurately quantified the average expression values of each E/M marker in every individual cell across the entire population of MDA-MB-231-CM cells, providing a robust comparison to populations of other sublineages. This approach has been used in many works to date, and it certainly has its limitations, like any other method. Thus, our study cannot exclude the possibility that different markers were expressed on different cells within a population. It is important to note that, alongside E/M marker expression, the hybrid E/M phenotype also has significant tumorigenic traits like clonogenic survival, stemness, metastasis, and resistance to treatment^40^. Our data on the presence of these traits (Figs. 1, 2, 3, 4, 5) in this cell subline clearly substantiated our confidence in the existence of a hybrid E/M phenotype in MDA-MB-231-CM cells. Thus, our findings challenges the traditional view of EMT as a binary process and aligns with recent studies suggesting that cancer cells can exist in intermediate EMT states^41^. We propose that the hybrid EMT state enhances cellular plasticity, enabling cells to swiftly adapt to varying microenvironments during metastasis and improve their resilience following chemotherapy treatment.

The differential cisplatin resistance observed between CM sublines of MDA-MB-231 and MCF7 cells highlights the complexity of drug response mechanisms in different breast cancer subtypes. Our observation of reduced apoptosis following CPT treatment comes in complete accordance with the recent study of Fanfone et al. (2022), where it was observed that cell undergoing confined migration have higher resistance to anoikis due to up-regulation of anti-apoptotic proteins^42^. Our findings indicate that repetitive exposure to cycles of serum deprivation and CM fosters a hybrid EMT phenotype, leading to a substantial increase in stage-specific embryonic antigen-4 (SSEA4) expression. Simultaneously, we observed a significant decrease in the proportion of CD133+ cells (Fig. 5). This transition may be responsible for the heightened resistance of MDA-MB-231CM cells to CPT-induced DNA damage. SSEA4 upregulation has been associated with increased drug resistance and cancer stem cell-like properties in various cancers^43^. The lack of similar changes in MCF7CM cells may account for their consistent susceptibility to the effects of CPT, highlighting the diverse adaptive responses seen among various breast cancer subtypes. The unexpected downregulation of CD133 in MDA-MB-231CM cells, contrasting with its upregulation in MCF7CM cells, challenges the universal applicability of CD133 as a cancer stem cell marker in breast cancer. This finding aligns with studies showing context-dependent expression and function of putative stem cell markers^44^. It suggests that the relationship between stem cell markers, EMT, and drug resistance may be more complex than previously thought and may vary depending on the cellular context.

The differential Ki67 expression patterns observed in CPT-treated CM cells provide further insight into their adaptive mechanisms. The reduction in Ki67 expression in MDA-MB-231CM cells suggests a potential quiescence-mediated survival strategy, which aligns with recent studies highlighting the importance of quiescence in cancer cell adaptation and drug resistance^45^. Conversely, the increase in Ki67 expression in parental MDA-MB-231 cells treated with CPT may represent an accelerated repopulation response, a phenomenon observed in various cancer types following chemotherapy^46^. Notably, the substantial increase in Ki67 expression induced by CPT in parental MDA-MB-231 cells (Fig. 9) corresponds directly with the marked accumulation of these cells in the S-phase of the cell cycle (Fig. 7) following CPT exposure. In the contrary, confined migration markedly diminishes CPT-induced Ki67 expression in MDA-MB-231CM cells, although there remains a noteworthy accumulation of these cells in the S-phase of the cell cycle as a result of CPT treatment (Fig. 7). This controversy underscores the idea that Ki67 is not simply a marker of cell proliferation status; it also serves as an indicator of heterochromatin reorganization which, in turn, likely contributes to the remodelling of gene expression^47,48^. If that is the case, then confined migration influences heterochromatin reorganization coupled to remodelling gene expression when responding to DNA-damaging agents such as CPT. Furthermore, changes in heterochromatin reorganization-gene expression may explain the survival advantage of cells such as MDA-MB-231CM when exposed to DNA-damaging drug treatment.

The consistent increase in nanobead uptake across both cell lines represents a novel finding with potential implications for cancer cell biology and drug delivery. Enhanced endocytic activity has been linked to increased malignancy and metastatic potential^49^. This increased uptake capacity could facilitate the internalization of signaling molecules or nutrients that support survival in challenging microenvironments during metastasis. Moreover, it may have implications for nanoparticle-based drug delivery strategies, suggesting that metastatic cells might be more susceptible to such approaches.

Our study highlights how confined migration alters cellular properties in breast cancer cell lines; however, we recognize that serum deprivation applied exclusively to test cells introduces a potential confounding variable. Serum deprivation can cause stress responses, change cell behavior, and lead to a quiescent state, while also helping to synchronize the cell cycle regardless of migration conditions. In solid tumors, fluctuating nutrient availability, including serum deprivation, is a common feature of the microenvironment. Thus, while serum deprivation may act as a confounding variable in controlled experimental systems, it also represents a physiologically relevant condition that influences tumor cell behavior. Despite these complexities, our findings suggest that confined migration induces complex and cell type-specific changes in breast cancer cells, affecting their stem-like properties, EMT status, drug resistance, and endocytic capacity. These findings contribute to our understanding of the adaptive mechanisms underlying metastasis and drug resistance in breast cancer. They also highlight the need for personalized approaches in cancer treatment, considering the heterogeneous responses of different cancer subtypes to environmental stresses. However, we cannot fully exclude the possibility that some of these effects are influenced by serum deprivation alone. Future studies incorporating serum-starved control systems will be necessary to disentangle these effects and confirm our conclusions.

### Limitations

This study has several limitations. Notably, the lack of a control system involving serum-starved cells limits our ability to definitively attribute observed effects solely to confined migration. Additionally, while we argue that serum deprivation reflects aspects of the tumor microenvironment, its role as a potential confounding variable cannot be excluded. These limitations underscore the need for further experimentation to isolate specific contributions of confined migration and nutrient deprivation. Future studies should also use single-cell transcriptomics and proteomics to understand better the traits of the hybrid E/M phenotype in individual cells treated with CM and serum deprivation.

## Methods

### Cell culture

MCF7 and MDA-MB-231 cell lines, obtained from the ATCC, were both kept in normal tissue culture media (NTCM) containing DMEM media supplemented with 10% FBS and 100 U/mL penicillin, and 100 U/mL streptomycin at $37\,^{\circ }$C and 5% CO_2_, and the MCF7 media was further supplemented with 0.01 mg/ml insulin (sigma). Cells were periodically tested for mycoplasma contamination

### Isolation of CM sublines of MDA-MB-231 and MCF7 cells

To generate the MDA-MB-231CM and MCF7CM, the CM-derived sublines, we employed a repetitive migration protocol using standard 6-well Boyden chambers ($\text {SPLInsert}^{\text {TM}}$Hanging, Cat #35206). In a series of three migration rounds, 5 × 10^5^ of either MDA-MB-231 or MCF7 cells, previously cultured under standard conditions and subjected to serum starvation for 24 hours, were introduced into the upper insert of a chamber. This insert, sealed at one end with an 8 µm pore size membrane, allowed the cells to migrate toward the lower chamber, which was filled with a complete growth medium containing 10% fetal calf serum (FCS) acting as a chemoattractant. In each migration round, MDA-MB-231 cells were permitted to migrate for 24 hours, whereas MCF7 cells were allocated a full 48 hours for their migration process. The extended migration period for MCF7 cells was necessary due to their significantly lower collective migration rates and confined migration rates compared to MDA-MB-231 cells. This approach ensured the collection of a sufficient population of migrated MCF7 cells for downstream cultivation. Following the migration round, the cells that successfully moved to the lower chamber were harvested and subjected to the next round of migration. This process was repeated for a total of three rounds of migration. After the third round of migration, the cells were subsequently cultured in NTCM (10% concentration of FBS) before being tested and analyzed. the resulting cells were designated as the CM sublines for each respective parental cell line.

### Anchorage-dependent clonogenic assay

MDA-MB-231 cells were cultured in six-well plates, while MCF7 cells were plated in 10 cm Petri dishes. For both cell lines, a single-cell suspension was prepared, and cells were plated at densities of 200, 300, and 500 cells per well. The cultures were then incubated for a total duration of two weeks, thereafter colony formation was monitored. By the end of the incubation period, colonies were fixed with methanol and stained with Giemsa stain to facilitate visualization. Colonies containing more than 50 cells were counted. Plating efficiency (PE) was calculated using the formula:

$$\begin{aligned} \text {PE}\% = \left( \frac{\text {Number of grown colonies}}{\text {Number of seeded cells}} \right) \times 100\% \end{aligned}$$

### Immuno-fluorescent assays

Single-cell High Content Imaging and Analysis of fluorescent labelling cells was used to measure the expression of E-cadherin (E-cad), N-cadherin (N-cad), and vimentin in cultured cells. Cells were grown in a 384-well plates (3000 cells/ well) with black walls for 24h in CO_2_ incubator at 37 °C. Following this, the cells were fixed with 4% paraformaldehyde for 15 minutes at room temperature (RT), and permeabilized with 0.1% Triton X-100 in phosphate-buffered saline (PBS) for 10 minutes. Thereafter, the non-specific binding was blocked with 5% bovine serum albumin (BSA) in PBS for 1 hour at room temperature.

The cells were then incubated overnight at $4\,^{\circ }$C with rabbit polyclonal anti N-cad (ELK Biotechnology, #ES2902), rabbit monoclonal anti N-cad (ABclonal Cat#A20798, RRID:AB_3107194), or mouse monoclonal anti-Vimentin (ELK Biotechnology, 1159) antibodies, diluted at a ratio of 1:200 in PBS containing 1% BSA (PBS-BSA). After primary antibody incubation, the wells were washed three times with PBS-0.05% Tween-20 (PBS-Tw) to remove unbound antibodies.

Subsequently, the cells were incubated with the following secondary antibodies diluted in PBS-BSA: goat anti-rabbit IgG H&L (Alexa Fluor 647, Abcam Cat#ab150079, RRID:AB_2722623) was used at a dilution of 1:1000 for E-cadherin and N-cadherin, and goat anti-mouse IgG H&L (Alexa Fluor 647, Abcam Cat#ab150115, RRID:AB_2687948) diluted to 1:1000 for vimentin. The incubation was carried out for 1 hour at RT in the dark, followed by washing three times with PBS-Tw. To visualize the cell nuclei, the wells were stained with Hoechst 33342 for 5 minutes before a final wash with PBS.

Fluorescence detected and quantified using $\text {CellInsight}^{\text {TM}}$ CX7 LZR Pro High Content Analysis Platform and built-in algorithms from ThermoFisher Scientific. The integrated fluorescence intensity data were obtained for E-cad, N-cad, and vimentin from each cell in each well.

### Cisplatin (CPT) sensitivity assay

To determine the IC50 concentration of CPT for the parental (P) and CM sublines of MDA-MB-231 and MCF7 cell lines, the sulforhodamine B (SRB) assay was employed. MDA-MB-231 and MCF7 cells were seeded in tissue-culture treated 96-well plates at a density 8000 and 3000 cells/well, respectively. Cells were cultured in a CO_2_ incubator at $37\,^{\circ }$C for 24 hours before being treated with a six-point dose increment of cisplatin (CPT) concentrations, ranging from 0 to 200 µM. This treatment was carried out over a total incubation period of 48 hours. After CPT treatment, 100 µL of 10% trichloroacetic acid (TCA) was introduced into the wells and incubated for one hour at $4\,^{\circ }$C to effectively precipitate the cellular proteins. Thereafter, the TCA solution was removed, and the plates were air-dried before adding 100 µL of a 0.057% SRB solution to each well. The plates were incubated at room temperature for 30 minutes to allow the dye to bind to cellular proteins. Unbound dye was washed away with 1% acetic acid, and the bound SRB dye was solubilized by adding 200 µL of 10 mM Tris base (pH = 10.5) to each well. The absorbance was measured at 510 nm (OD_510nm_) using CLARIOstar plate reader (BMG LABTECH, Germany). Percentage of cell survival was calculated based on the OD_510nm_ from PCT-treated wells relative to OD_510nm_ from control wells (untreated cells) multiplied by 100%, and IC50 values were determined by plotting the percentages of cell survival against CPT concentrations using nonlinear regression analysis algorithms of GraphPad Prism version 9.3.1 for Windows, GraphPad Software, San Diego, California USA, www.graphpad.com.

### Flow cytometry

To quantify the expression of CD133 and SSEA-4 antigens using flow cytometry, 500,000 cells/well of six-well plates were cultured in CO_2_ incubator at $37\,^{\circ }$C for 24h. Thereafter, the cells were trypsinized, washed and then fixed with 4% paraformaldehyde (PFA) for 15 minutes +4o C to preserve cellular morphology and antigenicity. After fixation, the cells were washed with PBS to remove excess PFA. For the detection of CD133, cells were incubated for 1h at RT with PE conjugated Anti-Human CD133/PROM1 Antibody (AntibodySystem Cat#SAA0050) diluted 1:200 with PBS-BSA in the dark. Following this, the cells were washed three times with PBS-Tw to remove unbound antibodies. For SSEA-4 detection, cells were similarly incubated with a primary antibody against SSEA-4 (MC813-70, Abcam Cat#ab16287, RRID:AB_778073) at a dilution of 1:200 for 1h at RT. After three washings with PBS-Tw, the cell-attached immune complexes were exposed to a secondary antibody goat anti-mouse IgG H&L (Alexa Fluor 647, Abcam Cat#ab150115, RRID:AB_2687948), diluted at 1:1000 in PBS- BSA, for an additional hour in the dark. Following incubation with the secondary antibody, the cells were washed again three times with PBS-Tw to eliminate any unbound antibodies. The cells were then resuspended in PBS for flow cytometric analysis. Flow cytometry was performed by recording at least 50,000 events using CytoFLEX flow cytometer (BECKMAN COULTER life sciences), and data were analyzed using $\text {FlowJo}^{\text {TM}}$ Software Version 10.10.0 Ashland, OR: Becton, Dickinson and Company; 2024. Available at: https://www.flowjo.com/ to quantify the expression levels of CD133 and SSEA-4 based on median fluorescence intensity (MFI).

### Apoptosis analysis

To measure apoptosis, an Annexin V-AF/PI apoptosis kit was utilized. Initially, 500,000 cells/well of six-well plates were cultivated in CO_2_ incubator at $37\,^{\circ }$C for 24 hours. CPT was added at concentrations of 80 µM for MDA-MB-231 cells and 15 µM for MCF7 cells. At 24 hours post-CPT treatment, the cells were harvested by trypsinization and washed with ice-cold PBS. Subsequently, the cells were resuspended in 1x binding buffer provided with the kit. For staining, 100 µL of the cell suspension was transferred to microcentrifuge tubes. Each tube received 5 µL of Annexin V-AF conjugate and was incubated for 15 minutes at room temperature in the dark to allow binding to phosphatidylserine on the cell membrane. Afterwards, 400 µL of 1x binding buffer was added to each tube. The propidium iodide (PI) was added at a final concentration of 5 µg/mL without prior washing. The samples were gently mixed and incubated for an additional 5 minutes at room temperature in the dark. The samples were kept on ice and measured within 1 hour of staining. Flow cytometry was performed using CytoFLEX flow cytometer (BECKMAN COULTER life sciences), and data were analyzed using $\text {FlowJo}^{\text {TM}}$ Software Version 10.10.0 Ashland, OR: Becton, Dickinson and Company; 2024. Available at: https://www.flowjo.com/ . Annexin V fluorescence was detected on the FITC channel, while PI fluorescence was recorded on the PC5.5 channel. Data acquisition involved analyzing at least 50,000 events per sample, allowing for quantification of viable (Annexin V−/PI−), early apoptotic (Annexin V+/PI−), late apoptotic (or necroptotic, Annexin V+/PI+), and necrotic (Annexin V−/PI+) cell populations.

### Cell cycle analysis

For cell cycle analysis, 500,000 cells/well of six-well plates were cultivated in CO_2_ incubator at $37\,^{\circ }$C for 24 hours. The next day, CPT was introduced at concentrations comparable to those used in the apoptosis assay. After a further incubation period of 48 hours, the cells were harvested by trypsinization and washed once with PBS to remove any residual media. Cells were fixed in 70% ethanol at $+4\,^{\circ }$C for 30 min, and then the samples were kept at $-20\,^{\circ }$C until the analysis was performed. Ethanol was removed and the cells were washed 3 times with PBS. Subsequently, the cells were resuspended in 100 µL of a propidium iodide (PI) solution at a concentration of 50 µg/mL, which also contained 10 µg/mL of RNAse A. They were allowed to incubate for one hour at RT in the dark before adding 400 µL of PBS followed by cytometry.

The stained cells were analysed using a CytoFLEX flow cytometer (BECKMAN COULTER life sciences), and data were analysed using $\text {FlowJo}^{\text {TM}}$ Software Version 10.10.0 Ashland, OR: Becton, Dickinson and Company; 2024. Available at: https://www.flowjo.com/ . Data acquisition involved analysis at least 50,000 events.

### Ki67 analysis

Cells were plated at a density of 3000 cells/well of tissue-culture treated 96-well plates. The following day, the media was replaced with fresh media containing CPT at concentrations of 80 µM for MDA-MB-231 and MDA-MB-231CM cells, and 15 µM for MCF7 and MCF7 CM cells. At 24-hour of CPT treatment, After the initial 24-hours of CPT treatment, the drug was removed, and the cells were washed with PBS before adding new fresh media. The cells were then incubated for an additional 48 hours to allow recovery before fixation. the cells were fixed with 4% paraformaldehyde (PFA) for 15 minutes at RT. Following fixation, the cells were washed three times with PBS to remove any residual PFA. All fixed cells were permeabilized using 0.1% Triton X-100 in PBS for 10 minutes at $4\,^{\circ }$C , followed by blocking with 5% BSA in PBS for 1 hour at RT.. The primary antibody specific for Ki-67 (Millipore Cat #MAB4190, RRID:AB_95092) diluted at 1:200 with PBS-BSA was incubated with the cells overnight at $4\,^{\circ }$C. After washing three times with PBS-Tw, the cells were incubated for 1 hour at RT in the dark with Goat Anti-Mouse IgG H&L (Alexa Fluor® 488) (Abcam Cat #ab150113, RRID:AB_2576208) antibodies, diluted at 1:1000 in PBS-BSA. Following incubation with secondary antibody, the cells were washed again three times with PBS-Tw. The nuclei were counterstained with Hoechst 33342 for 5 minutes before a final wash with PBS. Fluorescence images were captured using a $\text {CellInsight}^{\text {TM}}$ CX7 LZR Pro High Content Analysis Platform (ThermoFisher Scientific) fluorescence microscope.

### Analysis of nano-particle (NP) endocytosis

To assess cells’ uptake of NPs, 500,000 cells/well of six-well plates were cultivated in CO_2_ incubator at $37\,^{\circ }$C for 24 hours. The following day, cells in control wells were counted using an automated cell counter in order to calculate the amount of NPs to be added per cell. NPs (200 nm carboxylate-modified polystyrene beads, ThermoFisher Scientific, #F8811) were added at a quantity of 2000 NPs per cell to the corresponding experimental wells. Following incubation with the NPs for 1 hour at $37\,^{\circ }$C, the cells were washed once with PBS to remove unbound NPs, followed by another wash with PBS containing 0.02% sodium azid. The cells were then trypsinized and carefully laid over a 10% FBS cushion in conical microtubes. The cell pellets were collected by centrifugation at 300 $\times$ g in swinging-basket rotor for 5 minutes and aspiration of supernatants containing non-cell-associated beads. Subsequently, the pelleted cells were fixed with 4% paraformaldehyde (PFA) for 15 minutes at RT. After fixation, the cells were washed three times with PBS to remove any residual PFA. Then, the fixed cells were resuspended in PBS for flow cytometry analysis. The cells containing nano-beads were analyzed using a CytoFLEX flow cytometer (BECKMAN COULTER life sciences), and data were analyzed using $\text {FlowJo}^{\text {TM}}$ Software Version 10.10.0 Ashland, OR: Becton, Dickinson and Company; 2024. Available at: https://www.flowjo.com/ . Data acquisition involved analysis of least 50,000 events.

### Statistical approaches

Statistical analysis was performed using GraphPad Prism version 9.3.1 for Windows, GraphPad Software, San Diego, California USA, www.graphpad.com. Data are presented as mean ± standard deviation (SD), calculated from at least three independent repetitions of each experiment. Significance levels are marked with asterisks: *p< 0.05, **p < 0.01, ***p < 0.001, ****p < 0.0001.

## Data Availability

The data used to support the findings of this study are available from the corresponding author upon request.
